# Study of Hematological Parameters in Children Suffering from Iron Deficiency Anaemia in Chattagram Maa-o-Shishu General Hospital, Chittagong, Bangladesh

**DOI:** 10.1155/2014/503981

**Published:** 2014-10-21

**Authors:** Abu Syed Mohammed Mujib, Abu Sayeed Mohammad Mahmud, Milton Halder, Chowdhury Mohammad Monirul Hasan

**Affiliations:** ^1^Industrial Microbiology Research Division, Bangladesh Council of Scientific and Industrial Research, Chittagong 4220, Bangladesh; ^2^Department of Biochemistry and Molecular Biology, University of Chittagong, Chittagong 4331, Bangladesh

## Abstract

A total of 150 (30.61%) anemic patients out of 490 patients diagnosed to have iron deficiency anemia (IDA) have been selected for the first time in Bangladesh. For detailed study, blood samples from 150 anemic patients along with 25 controls were analyzed. Analysis of variance showed significant *P* value between mean platelet volume (MPV) in females (8.08 *μ*m^3^) and males (7.59 *μ*m^3^) (*P* < 0.05) in iron deficiency anemia patients. Besides, the value of white blood cells (WBC) in males (10946.08/cmm) was significantly higher than in females (9470.833/cmm) (*P* < 0.05). The significant correlation was observed among hemoglobin levels with hematocrits, hemoglobin with RBC, RBC with hematocrits, and MCV with MCH as well as MCH with MCHC. However, the negative correlation was observed between the hematological variables neutrophils and lymphocytes (*r* = −0.989). The common complaints we have found in the survey were weight loss 73.33%, attention problem 68%, dyspepsia 65%, decrease of appetite 72%, weakness 68%, diarrhea 65%, and headache 55% among IDA patients. ANOVA showed significant statistical difference in all the hematological and biochemical parameters. Analysis of variance test between anemias with only one of three biochemical parameters decreased and control showed that this group does not have iron deficiency.

## 1. Introduction

As the name implies, iron deficiency anemia is due to insufficient iron. Without enough iron, human body cannot produce enough hemoglobin; as a result, iron deficiency anemia causes tired and short breath. Worldwide, the most important cause of iron deficiency anemia is parasitic infection caused by hookworms, whipworms, and roundworms, in which intestinal bleeding caused by the worms may lead to undetected blood loss in the stool. These are especially important problems in growing children [1]. Malaria infections that destroy red blood cells (although the iron is recycled) and chronic blood loss caused by hookworms (where the iron is lost) contribute to anemia during pregnancy in most developing countries [2]. The principal cause of iron deficiency anemia in the developed countries is blood lost during menses in premenopausal women, which is not compensated by intake from food and supplements. Iron deficiency anaemia still remains the most common cause of anaemia not only in Bangladesh but also all over the world. According to the World Health Report, there are 1,788,600 people in this world suffering from iron deficiency anaemia. And iron deficiency anaemia is the foremost prevalent disease causing morbidity in the world [3]. Many surveys had been conducted in Bangladesh to know the prevalence of iron deficiency. A study of 114 healthy, socioeconomically privileged girls demonstrated that 24% of this group had no storage iron of and 42% of girls had suboptimal iron stores [4].

In a study of 85 men and 54 women in Finland, only traces of marrow iron were found in 4 to 7% of men, in 70% of women of 15 to 49 years of age, and in 23% of women of 50 years of age or older [5]. In a survey of 1105 Canadians, iron stores, judged by serum ferritin values, were greatly reduced in about 25% of children, 30% of pregnant women, and 3% of men [6]. In a study conducted by Looker AC. [7], it was found that 9% of toddlers aged up to 2 years, 9% to 11% of adolescent girls, and women of child bearing age were found to be iron deficient. Of these, iron deficiency anaemia was found in 3% and 2% to 5%, respectively. Also 7% of men over 50 years of age and 1% of young men and teen-age boys had iron deficiency [8]. In Pakistan, anaemia was present in 47% of children and 30% of the adult females [9]. Iron deficiency is particularly common in infants and pregnant women. The iron deficiency in women occurs most often during the reproductive years, whereas in men the incidence is relatively high in adolescent and low during young adulthood; it increases thereafter with advancing age. In infancy, the occurrence of iron deficiency is equal in both sexes. It is usually detected between the ages of 6 and 20 months; the peak incidence is at a younger age in infants born prematurely than in those born at term.

## 2. Methods

### 2.1. Study Design

A cross-sectional study was conducted from November 2010 to June 2011. The subjects were selected depending on the availability in the hospital ward. The hospital has facilities for iron deficiency anaemia diagnoses, treatment, and monitoring.

### 2.2. Study Subjects

A total of 150 subjects were included in this study. There was no specific predilection for race, religion, and socioeconomic status. The study subjects comprised the following two groups:

 group 1:
  - IDA male: 102 (subjects),
  - IDA female: 48 (subjects);
 group 2:
  - control male: 17,
  - control female: 8.

### 2.3. Ethical Consideration

Informed parental consent was taken before enrolling the children into the study. The procedure was fully explained to the parents and they were informed that if they wish they will be able to withdraw them from the study and it would not in any way hamper the treatment. Permission was also taken from the hospital authority, departmental head of the haematology laboratory, and biochemistry lab in order to undertake the study.

### 2.4. Inclusion Criteria and Exclusion Criteria

#### 2.4.1. Inclusion Criteria

Inclusion criteria are as follows:

1. all cases of suspected iron deficiency anaemia belonging the age group of 18 yrs,
2. all the patients having haemoglobin less than 11 gm/dL.

#### 2.4.2. Exclusion Criteria

Exclusion criteria are as follows:

1. patients previously transfused with blood within 120 days,
2. patients already on iron therapy.

### 2.5. Control and Development of Questionnaire

#### 2.5.1. Control

25 patients having haemoglobin within normal range were taken as control.

#### 2.5.2. Development of Questionnaire

A questionnaire was developed to obtain relevant information of demographic and socioeconomic data. The questionnaire also included anthropometric data, birth date, immunization history, past medical history, and clinical information. The questionnaire was coded and pretested before finalization. The questionnaire was both closed and open ended.

### 2.6. Blood Samples

Blood samples were collected by venepuncture using either the antecubital vein or the dorsal vein and dispensed into dipotassium EDTA anticoagulant bottles. Thereafter, informed consents were obtained from their parents/guardians and teachers. All haematological parameters were carried out by automatic methods. Adequate quality control measures were taken on each test procedure to ensure the reliability of the results. Haematological and biochemical investigations were done in haematology laboratory and biochemistry laboratory, respectively.

### 2.7. Serum Sample

Collect whole blood in a covered test tube. If commercially available tubes are to be used and should use the red toped tubes. These are available from Becton Dickinson (BD). BD's trade name for the blood handling tubes is Vacutainer. After collection of the whole blood, allow the blood to clot by leaving it undisturbed at room temperature. This usually takes 15–30 minutes. Remove the clot by centrifuging at 1,000–2,000 ×g for 10 minutes in a refrigerated centrifuge.

### 2.8. Biochemical Examination

#### 2.8.1. Serum Iron Estimation

Serum iron estimation was done with the help of the automated Dimension IRON method by Dade Behring Dimension Biochemistry Analyser.

#### 2.8.2. Total Iron Binding Capacity

TIBC estimation was done with the help of kit manufactured by Randox Laboratories LTD, UK.

#### 2.8.3. Serum Ferritin Assay

Serum ferritin assay was done with the help of ferritin serozyme kit manufactured by Biochem Immunosystems, Italy.

### 2.9. Hematological Examination

#### 2.9.1. Automated Blood Count (Complete Blood Count)

A complete blood count (CBC), also known as full blood count (FBC), was analyzed by the ABX PENTRA 60 which is a fully automated (Microprocessor controlled) haematology analyser used for the in vitro diagnostic testing of whole blood specimens. The ABX PENTRA 60 is able to operate either in CBC mode (12 parameters) or in CBC + 5DIFF mode (26 parameters). A scientist or lab technician performs the requested testing and provides the requested medical professional with the results of the CBC. The blood is well mixed (though not shaken) and placed on a rack in the analyzer. This instrument has many different components to analyze different elements in the blood. The cell counting component counts the numbers and types of different cells within the blood. The results are printed out or sent to a computer for review.

#### 2.9.2. HCT Measurement

The height of the impulse generated by the passage of a cell through the microaperture is directly proportional to the volume of the analyzed RBC. The haematocrit is measured as a function of the numeric integration of the MCV.

### 2.10. MPV Measurement

The MPV (mean platelet volume) is directly derived from the analysis of the platelet distribution curve.

### 2.11. Erythrocyte Sedimentation Rate (ESR) Measurement

We have followed Westergren method, collecting 2 mL of venous blood into a tube containing 0.5 mL of sodium citrate. It should be stored no longer than 2 hours at room temperature or 6 hours at 4°C. The blood is drawn into a Westergren-Katz tube to the 200 mm mark. The tube is placed in a rack in a strictly vertical position for 1 hour at room temperature, at which time the distance from the lowest point of the surface meniscus to the upper limit of the red cell sediment is measured. The distance of fall of erythrocytes, expressed as millimeters in 1 hour, is the ESR.

## 3. Results 

The diagnosis of iron deficiency anaemia was made only if all the three biochemical parameters like serum iron, serum ferritin, and percentage saturation of transferrin were below normal for the sex. By these criteria 150 (30.61%) out of 490 patients were diagnosed to have iron deficiency anaemia.

### 3.1. Age Distribution of IDA

**Figure 1:**
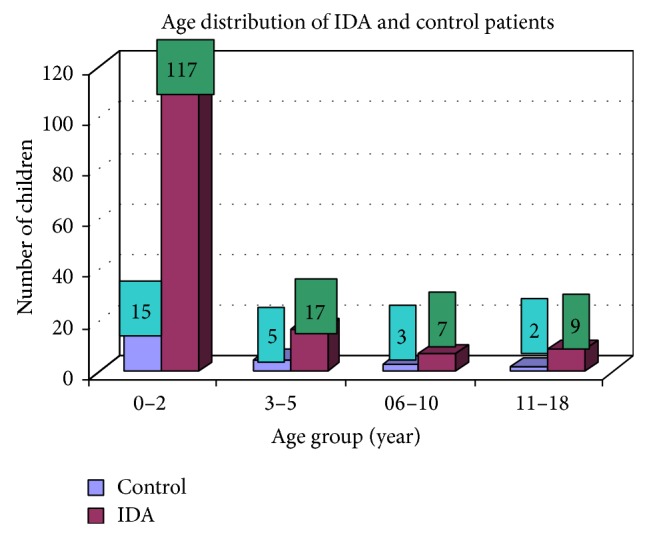
Showing age distribution of control and iron deficiency anaemia.

Out of 150 patients of IDA, 117 patients were in the age group of 0–2 years, 17 in the age group of 3–5 years, 7 in the age group of 6–10 years, and 9 in the age group of 11–18 years. The age distribution of iron deficiency anaemia and controls is shown in Figure 1.

### 3.2. Sex Distribution of IDA

**Figure 2:**
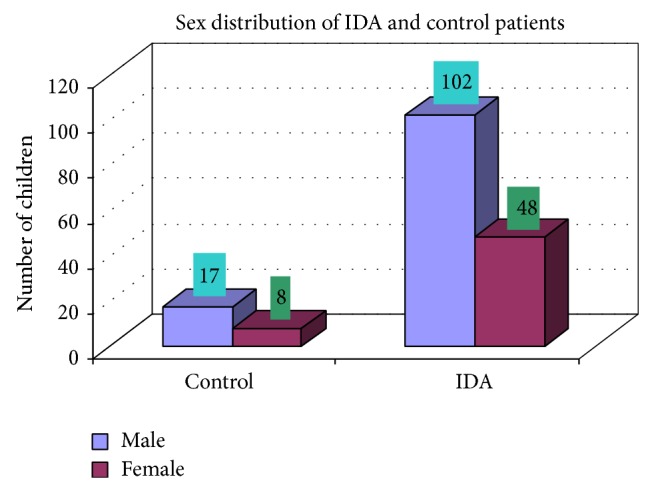
Showing sex distribution of IDA and control patients.

Of 150 patients with IDA patients, 102 were male and 48 were female and M : F ratio is 2.1 : 1. Of 25 controls, 17 were male and 8 were female and M : F ratio is 2.12 : 1 (Figure 2).

### 3.3. Biochemical Parameters

**Figure 3:**
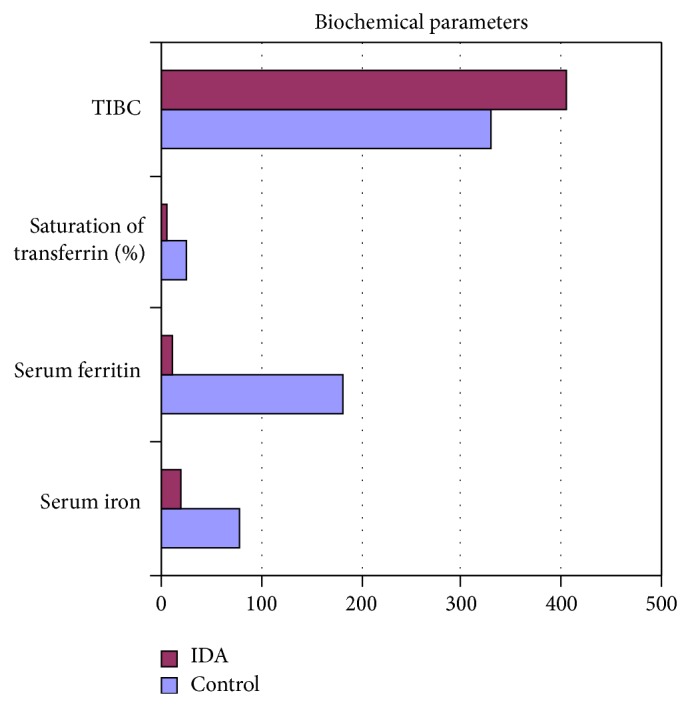
Showing biochemical parameters in control and IDA.

The mean value of serum iron in IDA was 20.85 g/dL, which is markedly less than that in control. The mean total iron binding capacity was greater in IDA (404.47 g/dL) than in control (Table 1). The mean percentage saturation of transferrin in IDA was found to be 5.73% that is markedly less than control. The mean serum ferritin in IDA was 9.94 ng/mL, which is less than control (Figure 3).

### 3.4. Complete Blood Count

**Figure 4:**
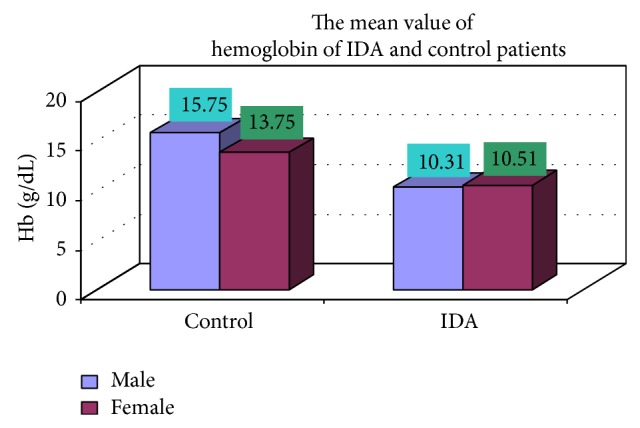
Showing the mean value of hemoglobin of control and iron deficiency anaemia.

**Figure 5:**
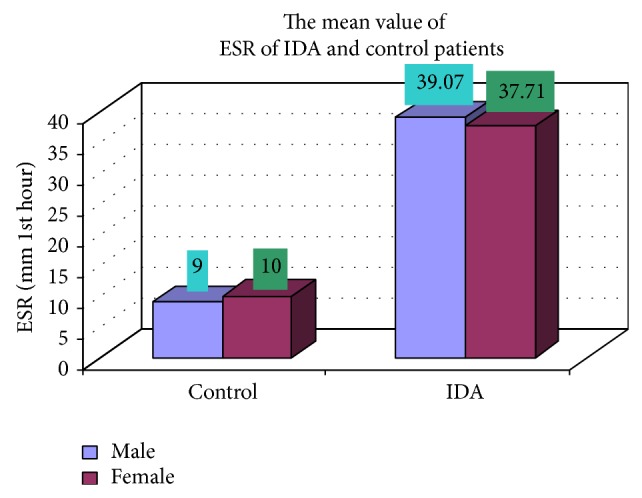
Showing the mean value of ESR of control and iron deficiency anaemia.

**Figure 6:**
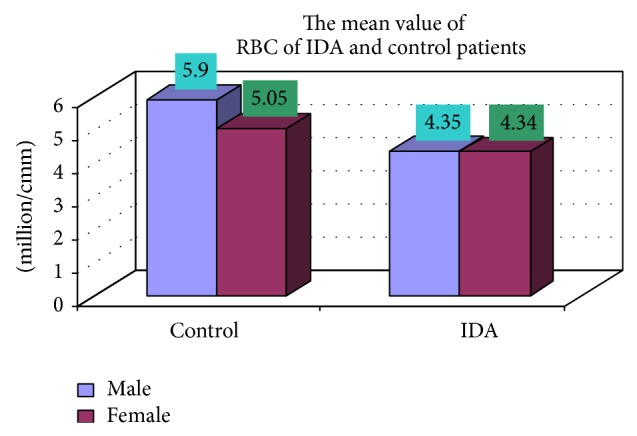
Showing the mean value RBC of control and iron deficiency anaemia.

**Figure 7:**
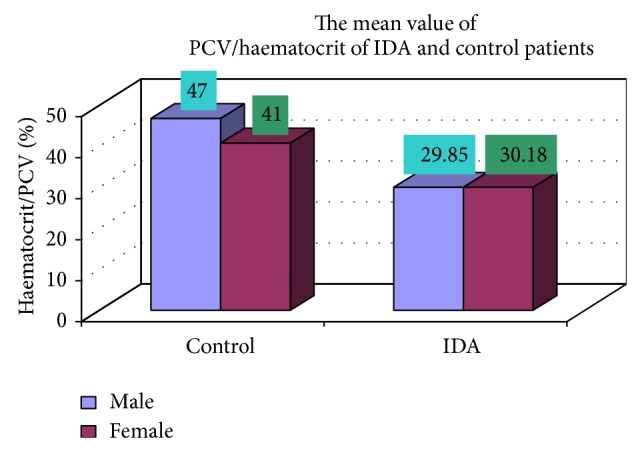
Showing PCV/hematocrit of control and iron deficiency anaemia.

**Figure 8:**
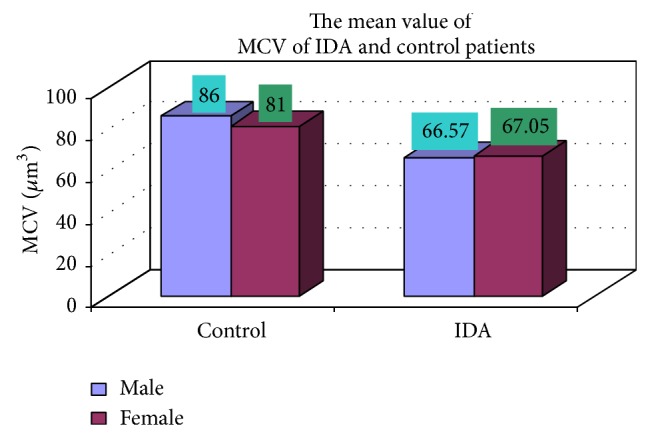
Showing the mean value MCV of control and iron deficiency anaemia.

**Figure 9:**
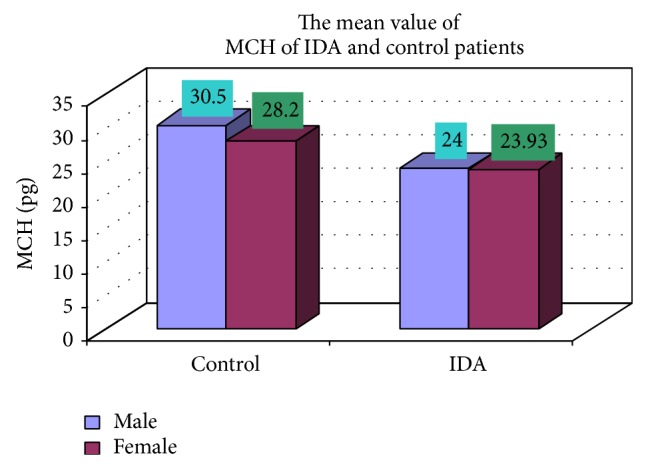
Showing mean value MCH of control and iron deficiency anaemia.

**Figure 10:**
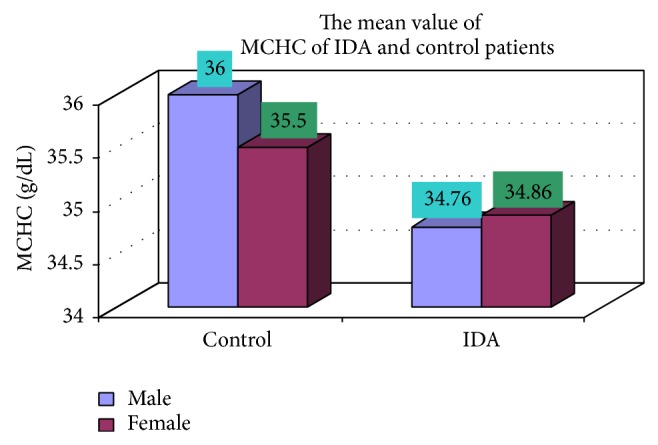
Showing the mean value MCHC of control and iron deficiency anemia.

**Figure 11:**
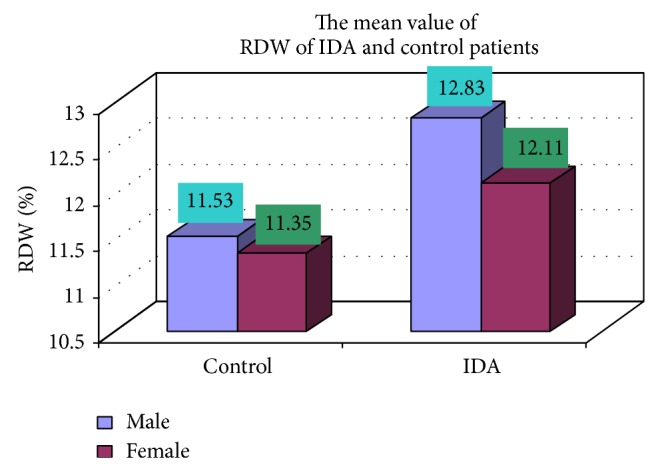
Showing the mean value RDW of control and iron deficiency anemia.

**Figure 12:**
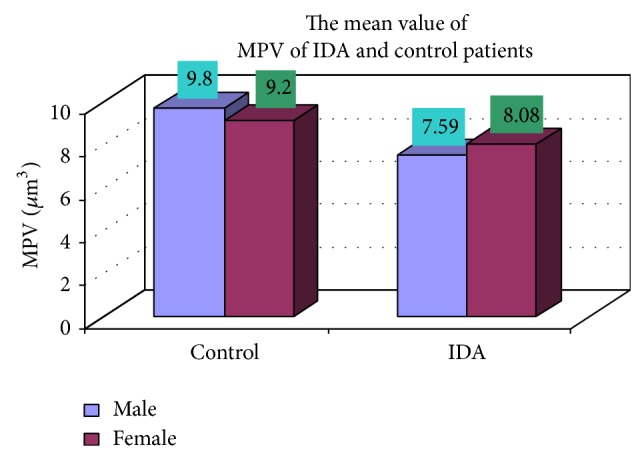
Showing the mean value of MPV of control and iron deficiency anaemia.

**Figure 13:**
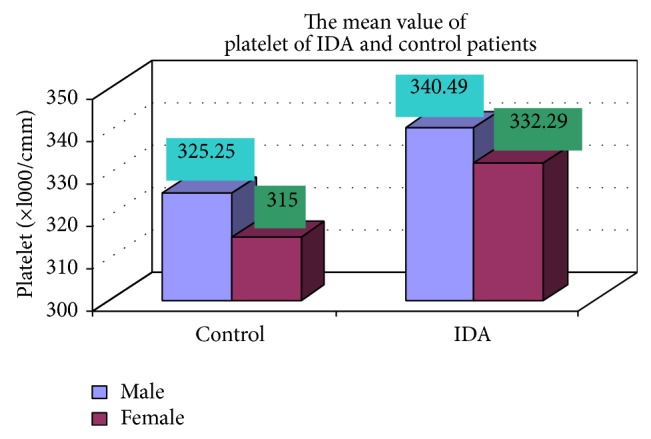
Showing mean value platelets of control and iron deficiency anemia.

**Figure 14:**
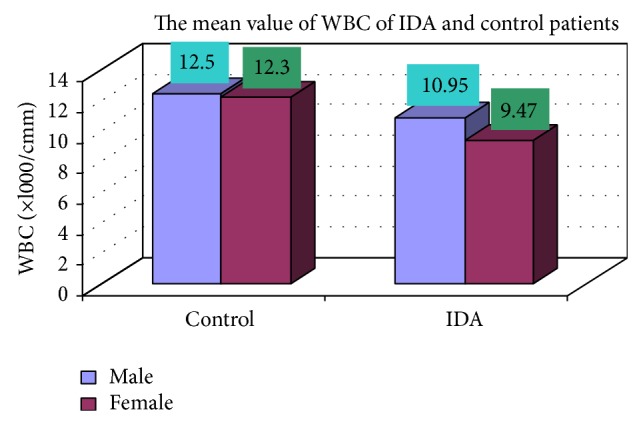
Showing mean value WBC of control and iron deficiency anaemia.

**Figure 15:**
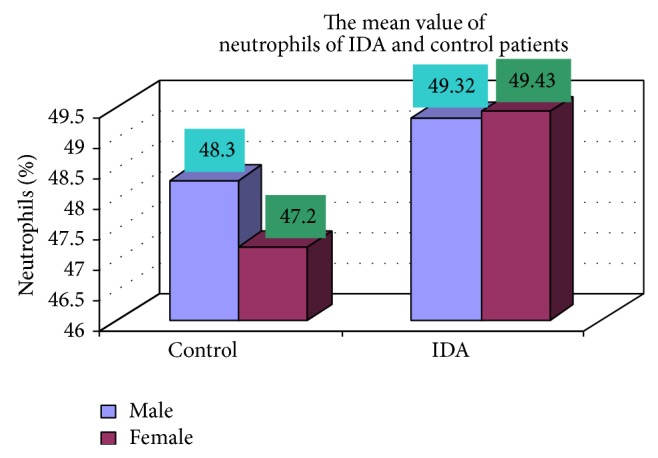
Showing mean value of neutrophils of control and iron deficiency anemia.

**Figure 16:**
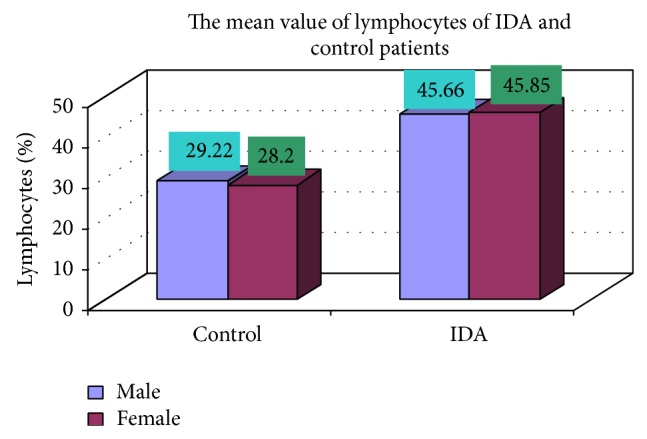
Showing means value of lymphocytes of control and iron deficiency anemia.

**Figure 17:**
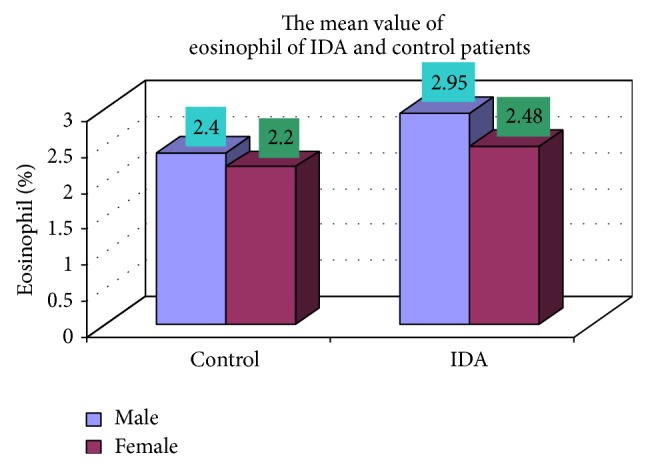
Showing mean value of eosinophil of control and iron deficiency anaemia.

**Figure 18:**
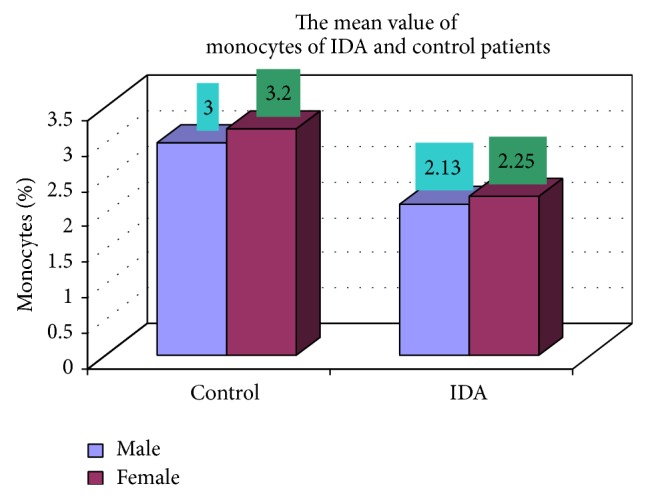
Showing the mean value of monocytes of control and iron deficiency anaemia.

The blood samples were collected from suspected anemic patient and control group, then complete blood count was conducted for the following hematological values like hemoglobin (Hgb), erythrocyte sedimentation rate (ESR), red blood cell (RBC), hematocrit (HCT), packed cell volume (MCV), mean corpuscular hemoglobin (MCH), mean corpuscular hemoglobin concentration (MCHC), red cell distribution width (RDW), mean platelet volume (MPV), platelet, white blood cell (WBC), neutrophil, lymphocyte, eosinophil, and monocytes. The results are represented graphically (Figures 4, 5, 6, 7, 8, 9, 10, 11, 12, 13, 14, 15, 16, 17, and 18).

### 3.5. Comparison of Hematological Parameters in Anemic Males and Females

We conducted complete blood count for various hematological values like hemoglobin (Hgb), erythrocyte sedimentation rate (ESR), red blood cell (RBC), hematocrit (HCT), packed cell volume (MCV), mean corpuscular hemoglobin (MCH), mean corpuscular hemoglobin concentration (MCHC), red cell distribution width (RDW), mean platelet volume (MPV), platelet, white blood cell (WBC), neutrophil, lymphocyte, eosinophil, and monocytes. The following (Table 2) is the comparative study of hematological values for IDA in males and females. Statistical analysis was carried out using Statistical Package for Social Sciences (SPSS) version 11.5, and an independent-sample *t*-test (*P* < 0.05) and one-way ANOVA test were used for comparison of hematological parameters. Results were considered to be statistically significant when the two-sided *P* value was less than 0.05 or (*P* < 0.05). ANOVA (analysis of variance) showed significant *P* value between mean platelet volume (MPV) in females (8.08 *μ*m^3^) and males (7.59 *μ*m^3^) (*P* < 0.05) in iron deficiency anemia patients (Tables 2, 3, 4, and 5). On the other hand, the value of white blood cells (WBC) in males (10946.08/cmm) was significantly higher than in females (9470.833/cmm), (*P* < 0.05).

The positive correlation among hematologic and biochemical variables was between hemoglobin and hematocrit (*r* = 0.851), hemoglobin and RBC (*r* = 0.659), RBC and hematocrit (*r* = 0.736), MCV and MCH (*r* = 0.806), and MCH and MCHC (*r* = 0.620), but not with other parameters.

The best negative correlation among hematologic variables was between neutrophils and lymphocytes (*r* = −0.989) but not with other parameters.

### 3.6. Clinical Features of IDA

**Figure 19:**
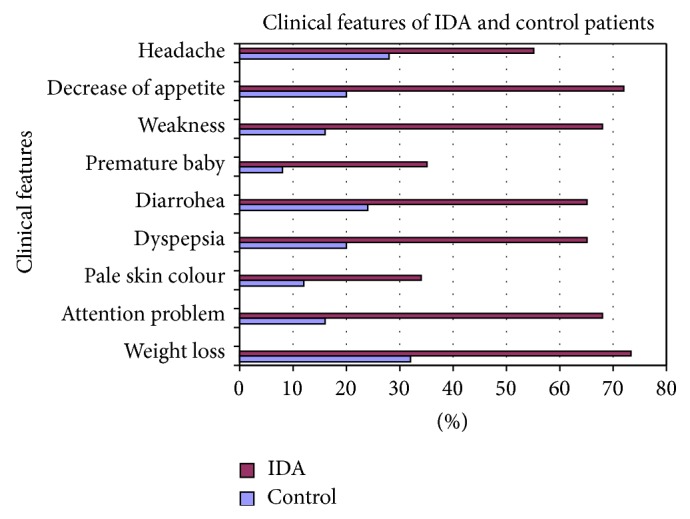
Showing clinical features of IDA and control patients.

**Figure 20:**
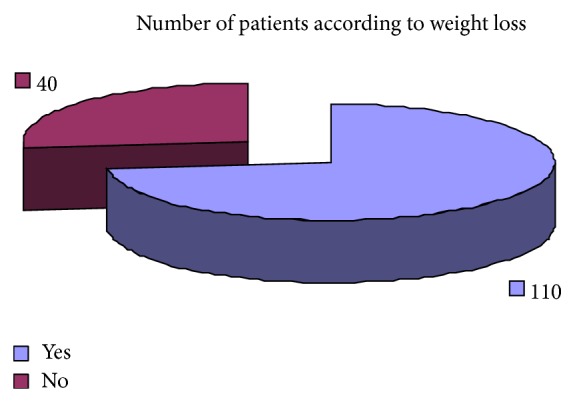
Showing the distribution of patients according to complaints of weight loss.

**Figure 21:**
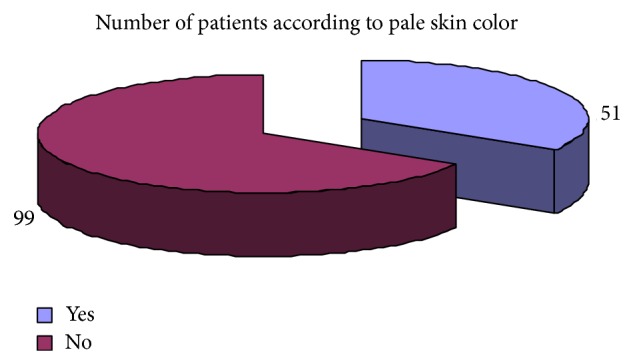
Showing the distribution of patients according to complaints of pale skin colour.

**Figure 22:**
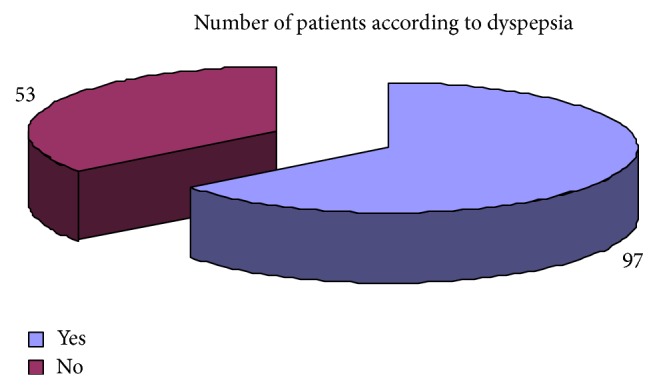
Showing the distribution of patients in accordance with complaints of dyspepsia.

**Figure 23:**
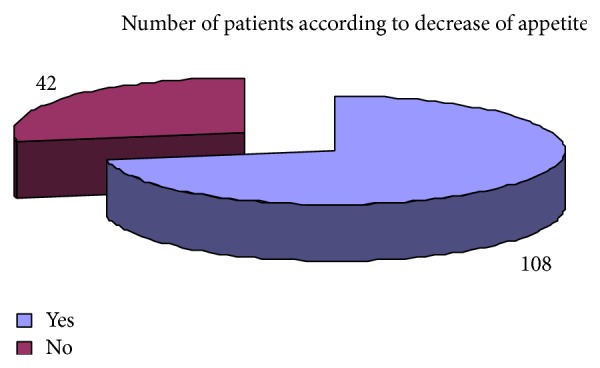
Showing the distribution of patients in accordance with complaints of decrease in appetite.

**Figure 24:**
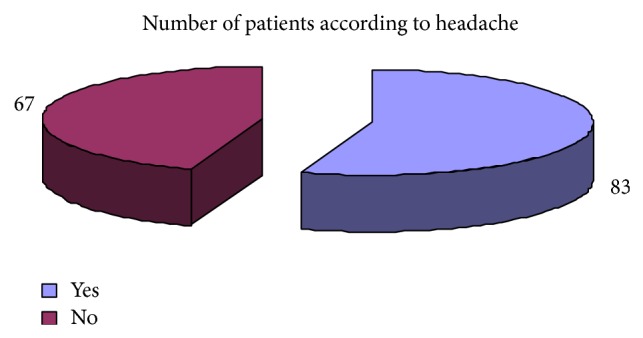
Showing the distribution of patients in accordance with complaints of headache.

Regarding the clinical presentation the most common complaints were weight loss 73.33%, attention problem 68%, dyspepsia 65%, decrease appetite 72%, weakness 68%, diarrhea 65%, and headache 55% (Table 6). Pale skin color and premature baby were the least common clinical presentation. The results regarding clinical complaints of iron deficiency have been shown graphically (Figures 19, 20, 21, 22, 23, and 24).

## 4. Discussion

In our study out of 150 IDA patients (102 males and 48 females) 78% were in age group between 0 and 2 years, 11.3% in age group between 3 and 5 years, 4.7% in age group between 6 and 10 years, and 6% in age group between 11 and 18 years. It should be noted that iron supplements and increased iron stores have recently been linked to maternal complications [10]. In contrast, our study showed that IDA is most commonly prevalent in the 0–2-year age group which includes 78% (male and female) and the sex ratio is also 2.12 : 1. These observations were taken from patients of medical OPD and ward. Moreover, hookworm infestation is more common in our context. Various researches have shown that hookworm is second most prevalent parasitosis in Nepal, incidence of which varies from 11% to 100% [11–13]. This could be the probable factor for the difference in the observations as stated before. Lower class (54%), middle class (31%), and upper class (15%) were mostly affected. These observations show that economically deprived and ignorant people are mostly affected. In clinical manifestations, weight loss, decrease of appetite, diarrhea, attention problems, and weakness were the most common complaints of the patients with IDA. Also, it is equally common among the anaemic individuals who were not iron deficient. Moreover, premature baby, pale skin color, dyspepsia, and headache were other complaints almost equally common among IDA and controls. These are symptoms of anaemia but not specifically concerned with IDA. These observations are in accordance with the previous reports of Elwood [12]. In our observation, the average MCV in IDA was 66.81 fl. Similarly the mean value of MCH was 23.97 pg, mean value of MCHC was 34.81%, and mean hemoglobin was 10.41 gm/dL in IDA. These observations are similar to the report by Bainton [14] and Finch C. A., which showed mean MCV to be 74 fl, mean MCH to be 20 pg, mean MCHC to be 28%, and mean hemoglobin to be 7.6 gm/dL in patients with IDA [15].

In the present study, the mean value of serum iron in IDA was 20.85 g/dL, which was significantly lower than control group (78.25 g/dL). In a study conducted in geriatric patients the mean was found to be 22.7 g/dL [16] which is almost similar to our result. The principal limitation of the serum iron determination is variability in the values [17], which may be due to both technical and physiologic factors [18] such as contamination of glassware and reagents with iron although the use of disposable, plastic equipment has reduced such contamination considerably. Among the biochemical tests, total iron binding capacity (TIBC) was increased in 42% out of the IDA patients (102 male and 48 female). The mean value of TIBC in IDA was 404.47*μ*g/dL, which was significantly higher than control group (329.42 *μ*g/dL). A study done in children IDA patients the mean TIBC was found to be 413.6 *μ*g/dL, which is also closer to our observation. Though increased value of TIBC indicates iron deficiency, the normal or even lower value may occur in iron deficiency anaemia [19]. So, TIBC was not included among the three biochemical parameters. Instead, in the present study, we have used percentage saturation of transferrin, the value of which does not overlap with the normal values and the values less than 16% occur in iron deficiency and anaemia of chronic diseases [20]. Values less than 5% are found only in iron deficiency. Statistical analysis was carried out using (*P* < 0.05); one-way ANOVA test was used for comparison of hematological parameters. ANOVA (analysis of variance) showed significant *P* value between mean platelet volume (MPV) in females (8.08 *μ*m^3^) and males (7.59 *μ*m^3^), (*P* < 0.05) in iron deficiency anemia patients. Besides, the value of white blood cells (WBC) in males (10946.08/cmm) was significantly higher than that in females (9470.833/cmm) (*P* < 0.05). ANOVA showed statistical significance in hematological and biochemical parameters between IDA and the control group and anaemia due to other diseases. It has also showed that the role of TIBC in diagnosing IDA cannot be overemphasized. ANOVA also showed that anaemia with decrease in any two of the three biochemical parameters might have iron deficiency while anaemia with decrease in one of the three biochemical parameters did not have iron deficiency. This problem must be overemphasized by public health system, because of too easy and available treatment and nutrition education for people. Our people do not know which foods to eat and which foods help their health.

## 5. Conclusion

Iron deficiency anaemia in children is still considered a major health problem all over the world. This is because of long term effects on mental and cognitive skills and on immunity and general physical wellbeing. More recently, iron deficiency is suggested to be related to DNA damage. This prospective study conducted in Chattagram Maa-O-Shishu General Hospital and Medical College, Agrabad, Chittagong, from November 2010 to June 2011 showed that iron deficiency anaemia is one of the most common anemias and in 30.61% of anaemic patients the diagnosis was confirmed by decrease in three biochemical parameters.

## Figures and Tables

**Table 1:** Mean biochemical parameters of IDA and control.

Biochemical parameters	Control	IDA
Serum iron (g/dL)	78.25	20.85
Serum ferritin (ng/mL)	180.33	9.94
% saturation of transferrin	25.51	5.73
TIBC (g/dL)	329.42	404.47

**Table 2:** Showing comparative hematological values for iron deficiency anemic males and females.

Parameters	Male	Female	*P* value
	mean ± SD	mean ± SD	
Hb (g/dL)	10.31 ± 1.46	10.51 ± 1.07	0.339
ESR (mm 1st hour)	39.07 ± 27.40	37.71 ± 25.68	0.768
RBC (million/cmm)	4.35 ± 0.59	4.34 ± 0.52	0.918
PCV/haematocrit	29.85 ± 3.75	30.18 ± 3.23	0.589
MCV (fl)	66.57 ± 5.32	67.05 ± 4.86	0.584
MCH (pg)	24.00 ± 2.93	23.93 ± 2.59	0.87
MCHC (g/dL)	34.76 ± 1.43	34.86 ± 0.98	0.626
RDW (%)	12.83 ± 2.08	13.11 ± 2.28	0.485
MPV (*µ*m^3^)	7.59 ± 0.85	8.08 ± 0.82	0.001
Platelet (×l000/cmm)	340.49 ± 82.57	332.29 ± 80.99	0.567
WBC (/cmm)	10946.08 ± 3786.32	9470.833 ± 2969.49	0.011
Neutrophil (%)	49.32 ± 16.27	49.43 ± 17.50	0.97
Lymphocytes (%)	45.66 ± 15.98	45.85 ± 17.21	0.947
Eosinophil (%)	2.95 ± 2.95	2.48 ± 1.60	0.208
Monocytes (%)	2.13 ± 1.05	2.25 ± 1.05	0.052

**Table 3:** Showing comparative hematological values between control and iron deficiency anemia (IDA) males and females.

Parameters	Control		IDA	
	Male	Female	Male	Female
	Number (mean)	Number (mean)	Number (mean)	Number (mean)
Hb (g/dL)	17 (15.75)	8 (13.75)	102 (10.31)	48 (10.51)
ESR (mm 1st hour)	17 (9)	8 (10)	102 (39.07)	48 (37.71)
RBC (million/cmm)	17 (5.9)	8 (5.05)	102 (4.35)	48 (4.34)
PCV/haematocrit	17 (47)	8 (41)	102 (29.85)	48 (30.18)
MCV (*µ*m^3^)	17 (86)	8 (81)	102 (66.57)	48 (67.05)
MCH (pg)	17 (30.5)	8 (28.2)	102 (24.00)	48 (23.93)
MCHC (g/dL)	17 (con)	8 (28.2)	102 (34.76)	48 (34.86)
RDW (%)	17 (11.53)	8 (11.35)	102 (12.83)	48 (13.11)
MPV (*µ*m^3^)	17 (9.8)	8 (9.2)	102 (7.59)	48 (8.08)
Platelet (×l000/cmm)	17 (325.25)	8 (315.00)	102 (340.49)	48 (332.29)
WBC (/cmm)	17 (12500.3)	8 (12300.2)	102 (10946.08)	48 (9470.833)
Neutrophil (%)	17 (48.3)	8 (47.2)	102 (49.32)	48 (49.43)
Lymphocytes (%)	17 (29.22)	8 (28.2)	102 (45.66)	48 (45.85)
Eosinophil (%)	17 (2.4)	8 (2.2)	102 (2.95)	48 (2.48)
Monocytes (%)	17 (3)	8 (3.2)	102 (2.13)	48 (2.25)

**Table 4:** Showing correlation among hematologic and biochemical variables in iron deficiency anemia patients.

	Hb	ESR (west)	RBC	PCV/Hct	MCV	MCH	MCHC	RDW	MPV
Hb	1	−0.281∗∗	0.659∗∗	0.851∗∗	0.441∗∗	0.329∗∗	0.159	−0.302∗∗	−0.020
ESR (west)		1	−0.252∗∗	−0.241∗∗	0.016	0.008	−0.064	0.032	−0.138
RBC			1	0.736∗∗	0.070	−0.118	−0.183∗	0.040	−0.090
PCV/hct.				1	0.448∗∗	0.289∗∗	0.125	−0.289∗∗	−0.121
MCV					1	0.806∗∗	0.410∗∗	−0.492∗∗	−0.085
MCH						1	0.620∗∗	−0.399∗∗	−0.017
MCHC							1	−0.366∗∗	0.084
RDW								1	0.004
MPV									1

^**^Correlation is significant at the 0.01 level (2-tailed). ^*^Correlation is significant at the 0.05 level (2-tailed).

**Table 5:** Showing correlation among hematologic variables in Iron deficiency anemia patients.

	Neutrophils	Lymphocytes	Eosinophils	Monocytes
Neutrophils	1	−0.989∗∗	−0.141	−0.035
Lymphocytes		1	0.035	−0.024
Eosinophils			1	−0.047
Monocytes				1

^**^Correlation is significant at the 0.01 level (2-tailed).

**Table 6:** Showing the frequency of clinical features of IDA and control patients.

Clinical features	Control	IDA
	Number (%)	Number (%)
Weight loss	8 (32%)	110 (73.33%)
Attention problem	4 (16%)	102 (68%)
Pale skin colour	3 (12%)	51 (34%)
Dyspepsia	5 (20%)	97 (65%)
Diarrhoea	6 (24%)	98 (65%)
Premature baby	2 (8%)	52 (35%)
Weakness	4 (16%)	102 (68%)
Decrease appetite	5 (20%)	108 (72%)
Headache	7 (28%)	83 (55%)
